# Discrepancies in publications related to HMB-FA and ATP supplementation

**DOI:** 10.1186/s12986-017-0201-7

**Published:** 2017-07-04

**Authors:** Jeremy A. Gentles, Stuart M. Phillips

**Affiliations:** 1 Department of Health Sciences, Armstrong State University, 11935 Abercorn St, Savannah, GA 31419 USA; 2 Department of Kinesiology, McMaster University, 1280 Main Street W, Hamilton, ON L8S 4K1 Canada

## Abstract

This letter addresses a number of discrepancies found in several publications related HMB-FA and ATP supplementation.

Our letter concerns the paper from Wilson et al. [1]. At the time of its publication in Nutrition and Metabolism, this paper [1] was the first of what are now three published articles registered with the clinical trial identifier: NCT01508338. The initial publication [1] now includes further papers by Wilson et al. [2] and a paper by Lowery et al. [3]. All are from the same study.

We focus here on an unexplained inconsistency between these papers [1–3]. First, the supplement protocols for this study are detailed on ClinicalTrials.gov [4], but differ from the published papers and are not consistent between the three aforementioned papers. Second, despite having the same control group in all three papers, the number of subjects in the control groups differ without explanation. Third, similarities between group means and standard deviations between all three papers illustrate an extraordinary level of homogeneity.

It is important to make clear that the three papers [1–3] are results from the same study, and the placebo group presented in all three papers is formed from the same subjects. This conclusion is supported by: 1) The record at ClinicalTrials.gov for this study, which lists only a single placebo group [4]; 2) In Lowery et al. [3], where the authors provide a retrospective analysis of all three papers and present data for only a single control group; and 3) From a personal email communication [5] with the principal investigator for all papers [1–3], where Dr. Jacob Wilson stated, “It was one large study divided into 3 papers which we mention in the HMB-ATP paper (reference [3] in this letter). So we had a control, HMB and HMB+ ATP group. For all 3 papers half of the subjects would be identical.”

Inconsistent descriptions of control groups between papers. According to the 2013 Wilson et al. article [1], subjects consumed the following supplement or placebo, “Prior to the study, participants were randomly assigned to receive either 400 mg per day of ATP disodium or maltodextrin (placebo)…” This description conflicts with the stated placebo protocol according to ClinicalTrials.gov [4]. Furthermore, in Wilson et al. [2], neither the placebo or HMB supplement protocols, match those described in Wilson et al. [1] or ClinicalTrials.gov [4]. Finally, Lowery et al. [3] is the only paper of the three, where the placebo and HMB + ATP supplement protocols match what is described on ClinicalTrials.gov [4], but this does not match the previous two papers. Could the authors explain how the reported placebo groups followed different supplement protocols, while simultaneously being in the same placebo group for the study?

Differing number of subjects in control groups between papers. In Wilson et al. [1], 3 subjects dropped due to injury leaving 11 in the ATP group and 10 in the placebo group. In Wilson et al. [2], 3 subjects dropped from the placebo group (2 due to injury and 1 for time commitment) and 1 from the HMB-FA group due to injury. In Lowery et al. [3], there were 9 in the placebo group and 8 in the HMB + ATP group with no explanation of why subjects dropped. Considering the evidence already provided establishing that all three publications are a result of the same study, and that the placebo group contained the same subjects, it would follow that the placebo group would be the same size in each publication. Equally troubling, is the absence of an explanation for dropouts and the original number of subjects in Lowery et al. [3]. Can the authors explain the discrepancies in the number of dropouts and reasons for dropout in Wilson et al. [1], Wilson et al. [2] and Lowery et al. [3]?

Extraordinary homogeneity between papers. Table 1 provides a side-by-side comparison of subjects’ age and measures of strength from Wilson et al. [1], Wilson et al. [2] and Lowery et al. [3]. These mean values represent the entire sample in each publication, not the means for each treatment groups. As can be seen, the samples in each publication have nearly identical means and SDs for age and 1RM strength relative to body mass for squat, bench press, and deadlift. The PI for the study communicated the following “…for any other group at the beginning of a study you can stratify subjects into groups so that body mass and strength are nearly identical. Thats [sic] the goal of any study to stratify not just so means are similar but also standard deviations.” [5] There are a number of issues with this explanation. Could the authors explain how this level homogeneity was achieved between all 3 publications with the absence of consistent subject/group matching, different sample sizes, and a different number of dropouts in placebo and treatment groups?

**Table 1:** Sample means and standard deviations

	Wilson et al. [1] Entire Group (*n* = 21)	Wilson et al. [2] Entire Group (*n* = 20)	Lowery et al. [3] Entire Group (*n* = 17)
Age (years)	21.6 ± 0.5 years	21.6 ± 0.5	21.7 ± 0.4
1RM squat^a^	1.7 ± 0.04	1.7 ± 0.04	1.7 ± 0.07
1RM bench press^a^	1.3 ± 0.04	1.3 ± 0.04	1.3 ± 0.05
1RM deadlift^a^	2.0 ± 0.05	2.0 ± 0.05	2.0 ± 0.06

^a^mean 1RM values are expressed relative to body mass
